# Protective Effects of* Streblus asper* Leaf Extract on H_2_O_2_-Induced ROS in SK-N-SH Cells and MPTP-Induced Parkinson's Disease-Like Symptoms in C57BL/6 Mouse

**DOI:** 10.1155/2015/970354

**Published:** 2015-12-21

**Authors:** Kanathip Singsai, Tarinee Akaravichien, Veerapol Kukongviriyapan, Jintana Sattayasai

**Affiliations:** Department of Pharmacology, Faculty of Medicine, Khon Kaen University, Khon Kaen 40002, Thailand

## Abstract

This study investigated the effects of* Streblus asper* leaf extract (SA) on reactive oxygen species (ROS) in SK-N-SH cell culture and on motor functions and behaviors in MPTP-treated C57BL/6 mice. SK-N-SH cell viability after incubation with SA for 24 h was measured by MTT assay. Intracellular ROS levels of SK-N-SH cells were quantified after pretreatment with SA (0, 200, 600, and 1000 *µ*g/mL) in the presence of H_2_O_2_ (300 *µ*M). Male C57BL/6 mice were force-fed with water or 200 mg/kg/day SA for 32 days. Intraperitoneal injection of MPTP was used to induce Parkinson's disease-like symptoms. Catalepsy, beam balance ability, olfactory discrimination, social recognition, and spontaneous locomotor activity were assessed on days 19, 21, 23, 26, and 32, respectively. In cell culture, SA at 200, 600, and 1000 *µ*g/mL significantly decreased ROS levels in H_2_O_2_-treated SK-N-SH cells. MPTP-treated C57BL/6 mice showed a significant change in all parameters tested when compared to the control group. Pretreatment and concurrent treatment with 200 mg/kg/day SA could antagonize the motor and cognitive function deficits induced by MPTP. The results show that SA possesses anti-Parkinson effects in MPTP-treated C57BL/6 mice and that reduction in ROS levels might be one of the mechanisms.

## 1. Introduction

Parkinson's disease (PD), causing movement disorders and nervous system problems in older adults, is the second most common neurodegenerative disease. Clinical features of PD include motor symptoms (bradykinesia, tremor, rigidity, and postural instability) and non-motor-related symptoms (olfactory deficits, autonomic dysfunction, depression, cognitive deficits, and sleep disorders) [1]. At present, the etiology and pathogenesis of PD are not fully understood and no animal model is able to reproduce all of its pathological features. In preclinical studies, many toxins are used to induce PD-like symptoms in animals. MPTP (1-methyl-4-phenyl-1,2,3,6-tetrahydropyridine) is one of the most successful of these agents. MPTP is a lipophilic compound that can cross the blood-brain barrier and, in glial cells, is metabolized by monoamine oxidase B into MPP^+^ (1-methyl-4-phenylpyridinium ion). MPP^+^ is then taken up by dopamine transporter and accumulated in mitochondria where it causes complex I inhibition and the generation of free radicals [2].

Levodopa remains the gold standard drug for relieving symptoms of PD [3]. Nevertheless, increasing interest has been devoted to the treatment or prevention of PD using herbal medicines [2].* Streblus asper* (SA) is a medicinal plant belonging to family Moraceae which inhabits various Asian countries, such as India, Sri Lanka, Malaysia, the Philippines, Southern China, and Thailand. Various parts of this plant are used in Ayurveda and other folk medicines [4]. It has also been shown to have many important pharmacological properties such as antimicrobial activity [5], anti-inflammatory effects [6], and antioxidant properties [7]. The aqueous extract of freeze-dried SA leaf is suggested to be a good potential source of natural antioxidants for preventing free radical-mediated oxidative damage [8]. Therefore, the purpose of this study was to investigate the effects of SA leaf extract on reducing the ROS in SK-N-SH cell cultures and on attenuating the abnormal behaviors in MPTP-treated male C57BL/6 mice.

## 2. Material and Methods

### 2.1. Plant Extract

Fresh leaves of SA were collected from local agricultural fields in Khon Kaen Province, Thailand. The fresh leaves were desiccated, crushed, and weighed. For aqueous extract preparation, the dried mashed powder was soaked in hot deionized water, filtered, and lyophilized. The percent yield of the SA extract was 12.69% of the dried leaves. The dry powdered extract was kept in airtight, light-protected containers at 2–4°C and dissolved in distilled water before being used.

### 2.2. Animals

Male C57BL/6 mice (6–8 weeks old, 25–30 g body weight) were purchased from National Laboratory Animal Center, Mahidol University, Thailand. The animals were housed in an animal room maintained at constant temperature (23–25°C) with a 12 : 12 h light : dark cycle. Food and water were available* ad libitum*. Mice were acclimatized to the laboratory room and handling for 1 week before the start of the experiments. All experiments were approved by the Animal Ethics Committee, Khon Kaen University, Thailand, record number AEKKU 12/2557, reference number 0514.1.12.2/17.

### 2.3. Determination of Free Radical Scavenging Activity by DPPH Assay

The free radical scavenging activity of SA was measured using stable radical 2, 2 diphenyl-1-picrylhydrazyl (DPPH) as previously described [9] with some modifications. In brief, the reactions (200 *µ*L), consisting of 100 *µ*L of 0.25 mM DPPH in ethanol and 100 *µ*L of various concentrations of SA extract (50, 100, 250, 500, and 1,000 *µ*g/mL), were plated in 96-well microtiter plates. Plates were incubated for 20 min in the dark, after which absorbance was measured at 540 nm. In this assay L-ascorbic acid (1–25 *µ*g/mL) was used as the reference standard. The reduction in absorbance was plotted against the concentration of the sample, and the IC_50_ values were determined using SigmaStat 3.5 software. The inhibitory percentage of DPPH was calculated according to the following equation:

(1) $$\begin{array}{c} Inhibition\left(\;\%\;\right)=\left(\;A_{0}-\frac{A_{1}}{A_{0}}\;\right)\times 100, \end{array}$$

where *A*_0_ is the absorbance of the control (DPPH alone) and *A*_1_ is the absorbance of reactions containing SA extract.

### 2.4. Cell Viability and Measurement of Intracellular ROS Levels in SK-N-SH Cell Cultures

SK-N-SH human neuroblastoma cells (HTB-11) (ATCC; Manassas, VA) were cultured in minimum essential medium (MEM) supplemented with 10% (v/v) fetal bovine serum, 15% (w/v) sodium bicarbonate, 0.1 mM nonessential amino acids, 1.0 mM sodium pyruvate, 100 *μ*g/mL streptomycin, and 100 units/mL penicillin G and maintained in a humidified incubator at 37°C with 5% CO_2_. Cell viability was evaluated by spectrophotometric analysis using MTT (3-(4,5-dimethylthiazol-2-yl)-2,5-diphenyltetrazolium bromide) (Sigma-Aldrich, St. Louis, MO). SA was diluted in MEM and added to the cells at final concentrations 0, 100, 200, 400, 600, 800, and 1000 *μ*g/mL. Cells were then incubated at 37°C for 24 h, after which MTT solution was added to each well at a final concentration of 500 *μ*g/mL. Formazan crystals formed by living cells were then dissolved in isopropanol and measured at 570 nm using a Multi-Detection Microplate Reader (BioTek Instruments, Inc., Winooski, VT).

Levels of intracellular ROS production were quantified using 2′,7′-dichlorodihydrofluorescein diacetate (DCFH-DA). Cells (3 × 10^4^ cells/mL) were seeded in 96-well plates and incubated for 24 h, after which DCFH-DA (50 *μ*M) was added to each well and incubation continued at 37°C in the dark for 45 min, followed by 45 min incubation with various concentrations (0, 200, 600, and 1000 *μ*g/mL) of SA. The cells were then treated with 300 *μ*M H_2_O_2_ (Merck Schuchardt OHG, Hohenbrunn, Germany) for 30 min. The fluorescence intensity of DCF was quantified with 485 nm excitation wavelength and 530 nm emission wavelength [10].

### 2.5. MPTP-Induced Parkinson's Disease-Like Symptoms in Mice

Male C57BL/6 mice were divided into 3 groups, 7–9 animals in each group. Animals were force-fed with either distilled water (as the control) or SA (200 mg/kg) once daily for the whole experimental period (32 days). On day 8, 45 min after oral treatment with SA, the animals were injected (i.p.) with distilled water or with MPTP. The MPTP regimen used was modified from previous reports [11–13]. In brief, mice were injected with eight doses of MPTP (each 10 mg/kg), with the total dose being 80 mg/kg. The first and second doses of MPTP were given on the first day 1 h apart. A single dose was injected once daily for another 6 days. On days 19, 21, 23, 26, and 32, catalepsy, beam balance ability, olfactory discrimination, social recognition, and spontaneous locomotor activity were assessed, respectively.

### 2.6. Catalepsy Test

Catalepsy, defined as a reduced ability to initiate movement and a failure to correct posture, was tested as described earlier [14]. Mice were subjected to the catalepsy test on day 19. The test is used to assess the muscle rigidity that is commonly seen in Parkinson's disease. On the test day, each mouse was positioned with its forepaws resting on a bar suspended above the floor. The length of time it remained in this position was recorded as time on bar. When the position was held for over 15 seconds, the test was stopped and time on bar was recorded as 15 sec.

### 2.7. Beam Balance Test

Balance and coordination of each mouse were assessed using the beam balance test on day 21. The apparatus used consists of a 120 cm long beam with a flat surface, 6 mm wide, resting 60 cm above the floor on two poles. A black box was placed at one end of the beam as the finish point. Nesting sawdust from home cages was placed in the black box to attract the mouse to the finish point [15]. On the test day, each mouse was placed at the start of the beam and the foot faults, defined as the number of slips by the forepaws and/or hind-paws from the horizontal surface of the beam, were counted by an investigator blind to drug treatment.

### 2.8. Olfactory Discrimination Test

Mice were subjected to the olfactory discrimination test on day 23. The task was used to assess their olfactory ability, degree of social interest, and perception of social novelty. The apparatus used was a plexiglass box divided into two equal compartments separated by an open door, one compartment with fresh sawdust (nonfamiliar compartment) and another with sawdust from the cage that the test mouse had occupied for the previous 3 days (familiar compartment). The task was based on the fact that rodents usually prefer places impregnated with their own odor (familiar compartments) [16]. On the test day, each mouse was placed in the box for 5 minutes and the time spent in each compartment was recorded.

### 2.9. Social Recognition Test

Mice were subjected to the social recognition test on day 26. This test was used to assess their short-term social memory [16]. The task consisted of two successive presentations (5 min duration for each presentation, 30 minutes apart), where a mouse unacquainted with the test animal was placed in the home cage of that animal. The time spent by the test animal investigating the unfamiliar mouse (nosing, sniffing, grooming, or pawing) was recorded. Between presentations, the unfamiliar mouse was removed and kept in an individual cage.

### 2.10. Spontaneous Locomotor Activity Test

Mice were subjected to an activity chamber on day 32. This task was used to assess general activity levels, locomotor activity, and exploratory habits [15]. The apparatus consisted of a square arena, 42 × 42 cm in size, surrounded by clear walls to prevent the test animal from escaping. The floor of the apparatus was divided into 36 squares of 7 × 7 cm with a 1 cm diameter hole in the center of each square. On test day, each mouse was placed on the center of the apparatus, and then locomotion (number of square crossings), grooming, nose poking, and rearing were recorded for 5 min using an AVH306 Network Video Recorder (Minics Communication Co., Ltd., Thailand).

### 2.11. Statistical Analysis

All results were presented as mean ± SE. Statistical comparisons between groups were analyzed with one-way analysis of variance (ANOVA) followed by post hoc Tukey's test. Results are considered to be statistically significant at *p* < 0.05.

## 3. Results

### 3.1. Free Radical Scavenging Activity of SA by DPPH Assay

Table 1 shows the antioxidant activities of the SA extract assessed using the DPPH radical scavenging assay. Percentage inhibition of DPPH activity by SA ranged from 2.75 ± 1.65% to 81.44 ± 0.34% at SA final concentrations between 25 and 500 *μ*g/mL. In this study, the IC_50_ values of ascorbic acid and SA were found to be 0.74 and 217.36 *μ*g/mL, respectively.

### 3.2. Effects of SA on Cell Viability and Intracellular ROS Levels in SK-N-SH Cells

**Figure 1:**
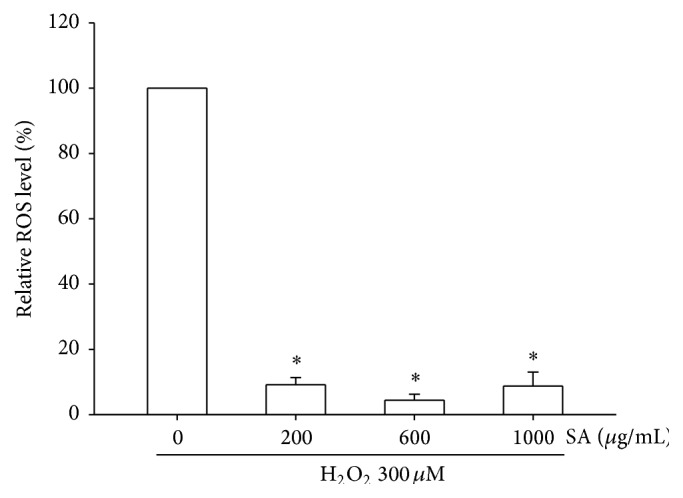
Effects of SA on intracellular ROS levels in SK-N-SH cells treated with H_2_O_2_ for 24 h with or without prior SA incubation. *∗* indicates significant difference when compared to the group treated only with H_2_O_2_.

In the MTT assay, SA at concentrations up to 1000 *µ*g/mL did not affect SK-N-SH cell viability (data not shown). The exposure of SK-N-SH cells to H_2_O_2_ at 300 *μ*M significantly increased intracellular ROS levels to about 150% of those in the untreated cells (data not shown). Preincubation of SK-N-SH cells with SA at 200, 600, or 1000 *µ*g/mL significantly decreased H_2_O_2_-induced intracellular ROS levels when compared to the H_2_O_2_-treated cells. The maximum effect was seen even at the lowest concentration of SA used (200 *µ*g/mL) (Figure 1).

### 3.3. Effects of SA on MPTP-Induced Motor Dysfunction

**Figure 2:**
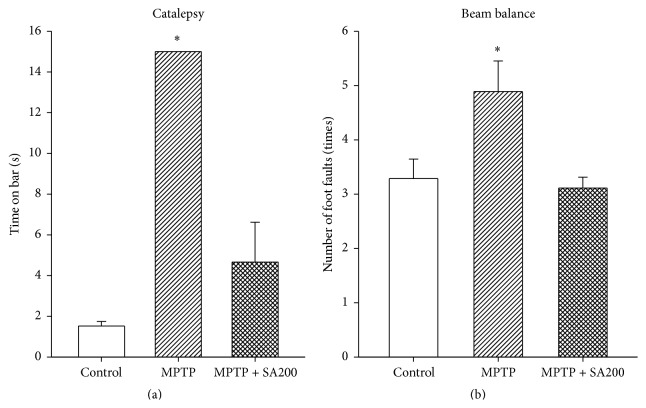
Effects of SA on MPTP-induced motor impairments. SA 200 mg/kg/day reduced time on bar (catalepsy time) relative to the MPTP-only group (a) and number of foot faults (b) induced by MPTP to a level comparable with the control. *∗* indicates significant difference from the control group.

On days 19 and 21 of the treatment, the animals were subjected to the catalepsy and beam balance tests, respectively. The mean time on bar for control animals was 1.52 ± 0.23 sec. Muscle rigidity or catalepsy was clearly observed in MPTP-treated mice and time on bar (>15 sec) was significantly increased when compared to the controls. Treatment with SA, 200 mg/kg/day, could antagonize MPTP-induced catalepsy and time on bar for the SA-treated group was 4.66 ± 1.96 sec (Figure 2(a)).

In the beam balance test, a significantly greater number of foot faults were observed in MPTP-treated mice relative to the controls (Figure 2(b)). SA significantly improved MPTP-induced motor deficits, as the numbers of foot faults were comparable to those in the control group.

### 3.4. Effects of SA Extract on MPTP-Induced Olfactory Deficit in Mice

**Figure 3:**
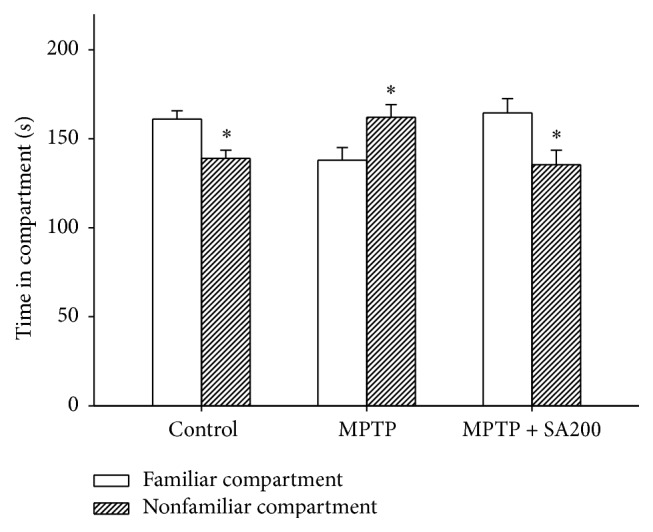
Effects of SA on MPTP-induced impairment of olfactory discrimination. MPTP-treated mice spent a significantly longer time in nonfamiliar than familiar compartments. SA 200 mg/kg/day reversed the pattern of olfactory discrimination induced by MPTP to the control pattern. *∗* indicates significant difference from familiar compartment of each treatment group.

Olfactory discrimination was tested on day 23 of the treatment. Control animals spent more time in the familiar than nonfamiliar compartments. In contrast, MPTP treatment caused an olfactory deficit in mice, as they spent more time in the nonfamiliar than familiar compartments (Figure 3). SA treatment reversed this effect of MPTP, with time spent in each compartment being similar to that in the control group.

### 3.5. Effects of SA Extract on MPTP-Induced Social Recognition Deficit in Mice

**Figure 4:**
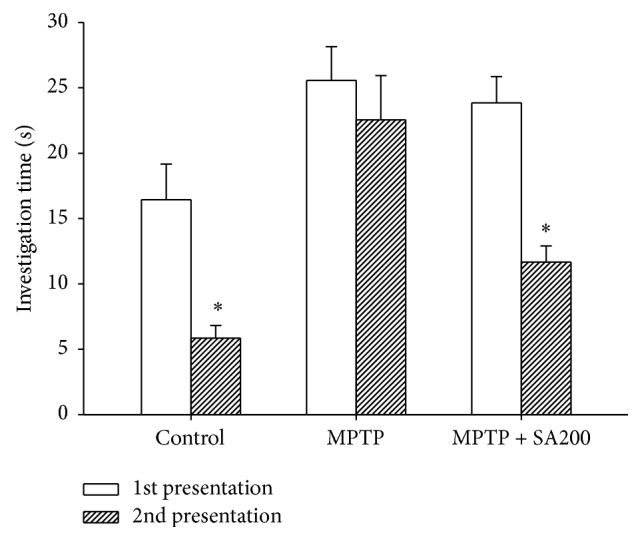
Effects of SA on MPTP-induced impairment of social recognition. MPTP-treated mice showed no difference in investigation time between the 1st and 2nd presentations. SA 200 mg/kg/day reversed the pattern of social recognition induced by MPTP to that seen in controls. *∗* indicates significant difference from the 1st presentation of each treatment group.

On day 26, social recognition tests were performed. The control animals showed a significant decrease in investigation time during the 2nd presentation of unfamiliar mice relative to the 1st presentation. Lack of difference in investigation times between the 1st and 2nd presentations in MPTP-treated mice indicated a social recognition deficit. Treatment with SA reversed this deficit, with investigation time during the 2nd presentation being significantly less than during the 1st presentation (Figure 4).

### 3.6. Effects of SA Extract on MPTP-Induced Abnormal Spontaneous Locomotor Activity in Mice

**Figure 5:**
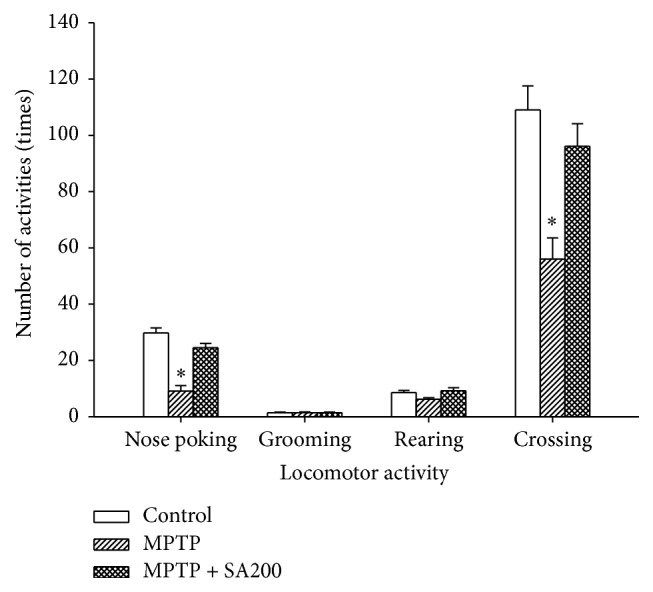
Effects of SA on MPTP-induced impairment of spontaneous locomotor activity. MPTP-treated mice showed a significant decrease in number of nose poking times and square crossings relative to the controls. SA 200 mg/kg/day reversed the effects of MPTP to the control pattern. *∗* indicates significant difference from the control.

Animals treated with MPTP showed a significant decrease in nose poking and square crossing activities, but not in grooming and rearing, relative to controls (Figure 5). SA reversed the effects of MPTP in both nose poking and crossing activities.

## 4. Discussion

We have shown that the aqueous extract of SA leaf has an antioxidant effect (DPPH assay), is not toxic to SK-N-SH cells, and reduces intracellular ROS level in SK-N-SH cells treated with H_2_O_2_. Moreover, the extract could antagonize the MPTP-induced Parkinson's disease-like symptoms in C57BL/6 mice, which included motor dysfunction, olfactory deficit, social recognition deficit, and changes in locomotor activities.

Evidence is increasingly showing that oxidative stress and inflammation contribute to the pathogenesis of neurodegenerative diseases including PD [17–22]. In the cell, reactive oxygen species (ROS) are generated primarily by mitochondria. Oxidative neuronal damage may be caused directly and/or indirectly through altered mitochondrial function [23]. MPTP is the gold standard agent used in toxin-based animal models of PD, replicating almost all of the PD hallmarks [24].

SA is a plant traditionally used in many countries. It is widely accepted that SA possesses analgesic, anti-inflammatory, and antioxidant effects and might have clinical applications in many disorders [4, 6–8, 25]. Recently, many lignans, flavonoids, and triterpenoids have been identified from SA leaves [26]. Some bioflavonoids identified from SA, such as kaempferol, myricetin, and ginkgetin, have been reported to have protective effects in animal PD models [27–29] and cell culture [30].

The neuroprotective effects of kaempferol and myricetin might be due to a reduction in oxidative stress [28, 30] and an inhibition of the mitogen-activated protein kinase (MAPK) kinase 4 (MKK4) [30], whereas ginkgetin acts by regulating iron homeostasis [29]. In the present study, active bioflavonoid components of SA might contribute to the effects on intracellular ROS generation induced by H_2_O_2_ in SK-N-SH cells and provide a protective effect in MPTP-induced PD model in mice. The data from this study suggest the potential of SA leaf extracts for prevention and treatment of PD.

## 5. Conclusion

In summary, we have investigated the effects of SA leaf extract on H_2_O_2_-induced ROS in SK-N-SH cells and MPTP-induced Parkinson's disease-like symptoms in C57BL/6 mouse. The results demonstrated that SA possesses the antioxidant activity and reverses the functional outcomes, including motor and cognitive functions, in MPTP-treated C57BL/6 mice. The present report suggests the possibilities that SA has an anti-Parkinson effect and can be one of the candidates for the development of a therapeutic agent for Parkinson's disease.

## Figures and Tables

**Table 1:** DPPH scavenging activities of SA.

SA concentrations (*µ*g/mL)	% inhibition (mean ± SEM)
25	2.75 ± 1.65
50	5.59 ± 6.73
125	19.03 ± 3.95
250	70.64 ± 1.16
500	81.44 ± 0.34

## References

[1] Glass C. K., Saijo K., Winner B., Marchetto M. C., Gage F. H. (2010). Mechanisms underlying inflammation in neurodegeneration. *Cell*.

[2] Li X.-Z., Zhang S.-N., Liu S.-M., Lu F. (2013). Recent advances in herbal medicines treating Parkinson's disease. *Fitoterapia*.

[3] Poewe W., Antonini A., Zijlmans J. C., Burkhard P. R., Vingerhoets F. (2010). Levodopa in the treatment of Parkinson's disease: an old drug still going strong. *Clinical Interventions in Aging*.

[4] Rastogi S., Kulshreshtha D. K., Rawat A. K. S. (2006). *Streblus asper* Lour. (Shakhotaka): a review of its chemical, pharmacological and ethnomedicinal properties. *Evidence-Based Complementary and Alternative Medicine*.

[5] Wongkham S., Laupattarakasaem P., Pienthaweechai K., Areejitranusorn P., Wongkham C., Techanitiswad T. (2001). Antimicrobial activity of *Streblus asper* leaf extract. *Phytotherapy Research*.

[6] Sripanidkulchai B., Junlatat J., Wara-aswapati N., Hormdee D. (2009). Anti-inflammatory effect of *Streblus asper* leaf extract in rats and its modulation on inflammation-associated genes expression in RAW 264.7 macrophage cells. *Journal of Ethnopharmacology*.

[7] Kumar R. B., Kar B., Dolai N. (2013). Antitumor activity and antioxidant role of *Streblus asper* bark against Ehrlich ascites carcinoma in Swiss albino mice. *Journal of Experimental Therapeutics and Oncology*.

[8] Ibrahim N. M., Mat I., Lim V., Ahmad R. (2013). Antioxidant activity and phenolic content of *Streblus asper* leaves from various drying methods. *Antioxidants*.

[9] Alothman M., Bhat R., Karim A. A. (2009). Antioxidant capacity and phenolic content of selected tropical fruits from Malaysia, extracted with different solvents. *Food Chemistry*.

[10] Sattayasai J., Chaonapan P., Arkaravichie T. (2013). Protective effects of mangosteen extract on H_2_O_2_-induced cytotoxicity in SK-N-SH cells and scopolamine-induced memory impairment in mice. *PLoS ONE*.

[11] Araki T., Kumagai T., Tanaka K. (2001). Neuroprotective effect of riluzole in MPTP-treated mice. *Brain Research*.

[12] Gibrat C., Saint-Pierre M., Bousquet M., Lévesque D., Rouillard C., Cicchetti F. (2009). Differences between subacute and chronic MPTP mice models: investigation of dopaminergic neuronal degeneration and *α*-synuclein inclusions. *Journal of Neurochemistry*.

[13] Sathiya S., Ranju V., Kalaivani P. (2013). Telmisartan attenuates MPTP induced dopaminergic degeneration and motor dysfunction through regulation of alpha-synuclein and neurotrophic factors (BDNF and GDNF) expression in C57BL/6J mice. *Neuropharmacology*.

[14] Esposito E., Impellizzeri D., Mazzon E., Paterniti I., Cuzzocrea S. (2012). Neuroprotective activities of palmitoylethanolamide in an animal model of Parkinson's disease. *PLoS ONE*.

[15] Curzon P., Zhang M., Radek R. J., Fox G. B., Buccafusco J. J. (2009). The behavioral assessment of sensorimotor processes in the mouse: acoustic startle, sensory gating, locomotor activity, rotarod, and beam walking. *Methods of Behavior Analysis in Neuroscience*.

[16] Prediger R. D. S., Aguiar A. S., Rojas-Mayorquin A. E. (2010). Single intranasal administration of 1-methyl-4-phenyl-1,2,3,6-tetrahydropyridine in C57BL/6 mice models early preclinical phase of Parkinson's disease. *Neurotoxicity Research*.

[17] Barnham K. J., Masters C. L., Bush A. I. (2004). Neurodegenerative diseases and oxidative stress. *Nature Reviews Drug Discovery*.

[18] Cappellano G., Carecchio M., Fleetwood T. (2013). Immunity and inflammation in neurodegenerative diseases. *American Journal of Neurodegenerative Disease*.

[19] Dauer W., Przedborski S. (2003). Parkinson's disease: mechanisms and models. *Neuron*.

[20] Hald A., Lotharius J. (2005). Oxidative stress and inflammation in Parkinson's disease: is there a causal link?. *Experimental Neurology*.

[21] Mosley R. L., Benner E. J., Kadiu I. (2006). Neuroinflammation, oxidative stress, and the pathogenesis of Parkinson's disease. *Clinical Neuroscience Research*.

[22] Przedborski S., Jackson-Lewis V., Vila M. (2003). Free radical and nitric oxide toxicity in Parkinson's disease. *Advances in Neurology*.

[23] Trushina E., McMurray C. T. (2007). Oxidative stress and mitochondrial dysfunction in neurodegenerative diseases. *Neuroscience*.

[24] Blesa J., Phani S., Jackson-Lewis V., Przedborski S. (2012). Classic and new animal models of Parkinson's disease. *Journal of Biomedicine and Biotechnology*.

[25] Afjalus S. M., Salahuddin M., Rahman M., Khatun A., Yasmin F. (2013). Investigation of analgesic and antioxidant of ethanolic extract of *Streblus asper* Lour. (Moraceae) leaf and bark. *International Research Journal of Pharmacy*.

[26] Li C., Huang C., Lu T. (2014). Tandem mass spectrometric fragmentation behavior of lignans, flavonoids and triterpenoids in *Streblus asper*. *Rapid Communications in Mass Spectrometry*.

[27] Filomeni G., Graziani I., De Zio D. (2012). Neuroprotection of kaempferol by autophagy in models of rotenone-mediated acute toxicity: possible implications for Parkinson's disease. *Neurobiology of Aging*.

[28] Li S., Pu X.-P. (2011). Neuroprotective effect of kaempferol against a 1-methyl-4-phenyl-1,2,3,6-tetrahydropyridine-induced mouse model of Parkinson's disease. *Biological and Pharmaceutical Bulletin*.

[29] Wang Y. Q., Wang M. Y., Fu X. R. (2015). Neuroprotective effects of ginkgetin against neuroinjury in Parkinson's disease model induced by MPTP via chelating iron. *Free Radical Research*.

[30] Zhang K., Ma Z., Wang J., Xie A., Xie J. (2011). Myricetin attenuated MPP^+^-induced cytotoxicity by anti-oxidation and inhibition of MKK4 and JNK activation in MES23.5 cells. *Neuropharmacology*.

